# Updated molecular phylogenetic data for *Opisthorchis* spp. (Trematoda: Opisthorchioidea) from ducks in Vietnam

**DOI:** 10.1186/s13071-017-2514-9

**Published:** 2017-11-21

**Authors:** Thanh Thi Ha Dao, Thanh Thi Giang Nguyen, Sarah Gabriël, Khanh Linh Bui, Pierre Dorny, Thanh Hoa Le

**Affiliations:** 1 National Institute of Veterinary Research, 86. Truong Chinh Street, Dong Da District, Hanoi, Vietnam; 2 Department of Biomedical Sciences, Institute of Tropical Medicine, Nationalestraat 155, B2000 Antwerp, Belgium; 3 Department of Virology, Parasitology and Immunology, Faculty of Veterinary Medicine, Ghent University, 133 Salisburylaan, B9820, Merelbeke, Belgium; 4 Department of Veterinary Public Health and Food Safety, Faculty of Veterinary Medicine, Ghent University, 133 Salisburylaan, B-9820 Merelbeke, Belgium; 5 Department of Parasitology, Faculty of Veterinary Medicine, Vietnam National University of Agriculture, Trau Quy, Gia Lam, Hanoi, Vietnam; 6 Department of Immunology, Institute of Biotechnology and Graduate University of Science and Technology, Vietnam Academy of Science and Technology, 18. Hoang Quoc Viet Rd, Cau Giay, Hanoi, Vietnam

**Keywords:** Mitochondrial gene, Ribosomal transcription unit, *Opisthorchis* sp. BD2013, Opisthorchiid, 18S rDNA, 28S rDNA, Phylogenetic analysis

## Abstract

**Background:**

An opisthorchiid liver fluke was recently reported from ducks (*Anas platyrhynchos*) in Binh Dinh Province of Central Vietnam, and referred to as “*Opisthorchis viverrini*-like”. This species uses common cyprinoid fishes as second intermediate hosts as does *Opisthorchis viverrini*, with which it is sympatric in this province*.* In this study, we refer to the liver fluke from ducks as “*Opisthorchis* sp. BD2013”, and provide new sequence data from the mitochondrial (mt) genome and the nuclear ribosomal transcription unit. A phylogenetic analysis was conducted to clarify the basal taxonomic position of this species from ducks within the genus *Opisthorchis* (Digenea: Opisthorchiidae).

**Methods:**

Adults and eggs of liver flukes were collected from ducks, metacercariae from fishes (*Puntius brevis*, *Rasbora aurotaenia*, *Esomus metallicus*) and cercariae from snails (*Bithynia funiculata*) in different localities in Binh Dinh Province. From four developmental life stage samples (adults, eggs, metacercariae and cercariae), the complete cytochrome *b* (*cob*), nicotinamide dehydrogenase subunit 1 (*nad*1) and cytochrome *c* oxidase subunit 1 (*cox*1) genes, and near-complete 18S and partial 28S ribosomal DNA (rDNA) sequences were obtained by PCR-coupled sequencing. The alignments of nucleotide sequences of concatenated *cob* + *nad*1 + *cox*1, and of concatenated 18S + 28S were separately subjected to phylogenetic analyses. Homologous sequences from other trematode species were included in each alignment.

**Results:**

Phylogenetic trees were inferred from concatenated (*cob* + *nad*1 + *cox*1) nucleotide sequences and combined 18S + 28S nucleotide sequences of five *Opisthorchis* sp. BD2013 samples and additional reference taxa. Both trees demonstrated the anticipated clustering of taxa within the superfamily Opisthorchioidea, the paraphyly of the genus *Opisthorchis* and the sister-species relationship of *Opisthorchis* sp. BD2013 with *O. viverrini*.

**Conclusions:**

While it is likely that *Opisthorchis* sp. BD2013 is distinct from *O. viverrini*, it is clearly a sister taxon of *O. viverrini* within the limited number of *Opisthorchis* species for which appropriate sequence data are available. The new sequences provided here will assist the diagnosis and the taxonomic clarification of the opisthorchiid species.

## Background

The family Opisthorchiidae (Digenea: Opisthorchioidea) consists of 33 genera considered valid including the genera *Opisthorchis* and *Clonorchis*, in which *O. viverrini, O. felineus* and *C. sinensis* are known to infect humans [1]. Humans become infected by eating uncooked cyprinoid fish containing metacercariae. *Opisthorchis viverrini* has been reported in Central Vietnam, where Binh Dinh and Phu Yen Provinces are highly endemic for human opisthorchiasis [2–4].

In 2013, Dao et al. [5] found adults of an opisthorchiid species in ducks (*Anas platyrhynchos*) in areas of Binh Dinh Province where there are many human opisthorchiasis cases. This parasite was then given the working name “*Opisthorchis viverrini*-like”, because of its close similarity to *O. viverrini* [5, 6]. Subsequently, there has been a debate about the identity of this worm. Nawa et al. [7] argued that the duck liver fluke not be *O. viverrini*, but is most likely *O*. *parageminus* that was previously reported from ducks in Vietnam [8–10]. However, Dorny et al. [11] considered that their “*Opisthorchis viverrini*-like” species exhibited some morphological differences from *O. parageminus*. We now propose to use the working name “*Opisthorchis* sp. BD2013” instead of the earlier “*Opisthorchis viverrini*-like”.

Molecular phylogenetic/systematic studies are excellent aids for taxonomy [12–15]. Such studies require homologous sequences from as many taxa as possible within the group of interest. In the genus *Opisthorchis*, a number of genetic markers from complete mitochondrial sequences and the nuclear ribosomal transcription units including, ITS1, ITS2, 18S rDNA and partial 28S rDNA have been generated for *O. viverrini*, *O. felineus* and *Clonorchis sinensis*. These genetic markers have greatly contributed to molecular diagnostic, epidemiological, phylogenetic and evolutionary studies of the species in Opisthorchiidae and trematodes [3, 13, 16–19]. However, *Opisthorchis* is a very large genus [7], and molecular data are available for only a few species. Moreover, given difficulties with the morphological taxonomy within the genus, it is not always certain that names assigned to samples are accurate. The only molecular data claimed to be from *O. parageminus* consist of two sequences recently deposited in GenBank (accession numbers KX258656, KX258657) by Nguyen and Nguyen (otherwise unpublished data). Although their worms came from ducks in Vietnam, no information is available on the morphological basis for the identification. Both of these sequences (mitochondrial partial mt *cox*1 and nuclear ribosomal ITS2) are very similar to earlier sequences available for *Opisthorchis* sp. BD2013 published by [5]. Here, we provide additional mitochondrial sequences, i.e. complete cytochrome *b* (*cob*), nicotinamide dehydrogenase subunit 1 (*nad*1) and cytochrome *c* oxidase subunit 1 (*cox*1) genes, and near-complete 18S rDNA and partial 28S rDNA sequences in an effort to better resolve the affinities of *Opisthorchis* sp. BD2013 within the family Opisthorchiidae and the superfamily Opisthorchioidea.

## Methods

### *Opisthorchis* sp. BD2013 samples collected from the field

Adult specimens and eggs of *Opisthorchis* sp. BD2013 were collected from naturally infected domestic ducks (*Anas platyrhynchos*) originating from 4 localities (Phu Cat, Phu My, An Nhon and Tuy Phuoc Districts) in Binh Dinh Province of Central Vietnam [6, 20] (Table 1). Each adult worm, unstained or stained with acetic carmine, was morphologically identified by light microscopy [5]. Up to three adult worms from each locality were individually fixed in 70% ethanol, and one or two worms from each locality were separately subjected to genomic DNA extraction and molecular analysis.

**Table 1:** List of field samples used in this study, their geographical collection site in Binh Dinh province and their hosts

Life-cycle stage	Site collected (district)	Host	Scientific name	Sample abbreviation for use in this study
Adult worm	Phu Cat	Duck	*Anas platyrhynchos*	*Opisthorchis* sp. BD2013-PC6aduBD
Adult worm	Phu My	Duck	*Anas platyrhynchos*	*Opisthorchis* sp. BD2013-PM10aduBD
Adult worm	An Nhon	Duck	*Anas platyrhynchos*	
Adult worm	Tuy Phuoc	Duck	*Anas platyrhynchos*	
Metacercariae	Phu My	Fish	*Puntius brevis*	*Opisthorchis* sp. BD2013-PCmetaBD
Metacercariae	Phu My	Fish	*Rasbora aurotaenia*	
Metacercariae	Phu My	Fish	*Esomus metallicus*	
Cercariae	Phu My	Snail	*Bithynia funiculata*	*Opisthorchis* sp. BD2013-PCcercaBD
Eggs	Phu My	Duck	*Anas platyrhynchos*	*Opisthorchis* sp. BD2013-PCeggBD

Fishes (harbouring metacercariae) and snails (shedding cercariae) were collected from My Tho Lake in the lowlands of Binh Dinh Province [20]. Infected fishes were identified as *Puntius brevis*, *Esomus metallicus*, *Rasbora aurotaenia*, and the snail as *Bithynia funiculata* [20] (Table 1). For molecular analysis, metacercariae and cercariae were individually fixed in RNAlater™ buffer (Qiagen, Texas, USA) at 4 °C. Individual parasites from each intermediate host and each locality were used for extraction of DNA and molecular study.

Eggs were individually collected from the gallbladder of naturally infected ducks by washing and centrifuging the bile ten times in normal saline (0.9% NaCl), then three times in phosphate buffered saline (PBS) before storage at -20 °C until use (Table 1).

### Genomic DNA extraction and primers

Total genomic DNA was extracted from individual adults, metacercariae, cercariae or pooled eggs (approximately 2000–3000 eggs) using the GeneJET™ Genomic DNA Purification Kit (Thermo Fisher Scientific Inc., MA, USA), according to the manufacturer’s instructions. A slight modification applied for eggs was to increase the incubation period by 3–4 h after enzymatic lysis. Genomic DNA was eluted in 50 μl of the elution buffer provided in the kit and stored at -20 °C. The DNA concentration was estimated using a GBC UV/visible 911A spectrophotometer (GBC Scientific Equipment Pty. Ltd., Braeside, Australia) and diluted to a working concentration of 50 ng/μl (about 10 ng/μl for DNA from eggs). From this genomic DNA, 2–3 μl was used as template in a PCR of 50 μl volume.

Primers used both for amplification and sequencing of the mitochondrial and nuclear ribosomal genes are listed in Table 2. The primer pair OACOBF/OACO1R amplified approximately 7.8 kb of mtDNA. Based on the sequence obtained from this amplicon, three primer pairs specific for the individual target protein-coding genes were designed. Primer pairs OACOBF/OACOBR, OAND1F/OAND1R, OACO1F/OACO1R amplified complete *cob*, *nad*1 and *cox*1 genes, respectively. The primer pairs U18SF/U18SR were used for obtaining major fragments of ribosomal 18S and U28SF/U28SR for 28S, respectively [12]. Additional internal primers were designed and used as needed (Table 2).

**Table 2:** Primers for amplification and sequencing of the mitochondrial protein-coding and nuclear ribosomal genes used in this study

Primer name	Sequence (5′–3′)	Target gene	Amplicon by PCR	Length of sequence (bp)	Reference
OACOBF	AGCCGGAGAGTCATTGTGTG	*cob*	1.4 kb	1110	This study
OACOBR	TGAATCCCACAACCGCGTTA				
OACOBR2^a^	TACGTTGAAGGACGGGTTGG				
OAND1F	CGTGTGGTGGGGCAAGATAG	*nad*1	1.2 kb	903	This study
OAND1R	CCACACAGCCTTCTCAAGGT				
OACO1F	GAGGGTTACGTGGGTTGGAG	*cox*1	1.8 kb	1551	This study
OACO1R	CAACCCTACTAAGCACCACAGC				
OACO1R2^a^	GGATCCCAAAAACGCTCACG				
U18SF	GCGAATGGCTCATTAAATCAGC	18S	1.8 kb	~ 1790	[12]
U18SR	GGAACCAATCCGAGGACCTTGC				
NS2F^a^	GCAAGTCTGGTGCCAGCAGCC				
U28SF	CTAACAAGGATTCCCTTAGTAAC	28S	1.3 kb	~ 1100	[12]
U28SR	GTCTTTCGCCCCTATACTCAC				

*Abbreviations*: *F* forward, *R* reverse

^a^Internal primer used for sequencing

### Amplification of mitochondrial and ribosomal genes

#### The 7.8 kb mt genomic region

Long PCR reactions were prepared using 25 μl of Fusion High-Fidelity PCR Master Mix (2×) (Thermo Fisher Scientific Inc., Waltham, MA, USA) and 2 μl of each primer (10 pmol/μl), 2 μl DNA template of the adult sample (50 ng/μl), 2 μl DMSO (dimethyl sulfoxide) and 17 μl H_2_O up to a final volume of 50 μl. All PCRs were performed in an MJ PTC-100 thermal cycler with initiation at 98 °C for 30 s, followed by 35 cycles consisting of denaturation for 10 s at 98 °C, annealing at 56 °C for 30 s, extension at 72 °C for 6 min.

#### Individual mt and ribosomal DNA genes

PCR reactions of 50 μl were prepared using 25 μl of DreamTaq PCR Master Mix (2×) (Thermo Fisher Scientific Inc., Waltham, MA, USA), 2 μl of each primer (10 pmol/μl), 2 μl DNA template (50 ng/μl for adults; 50 ng/μl for metacercariae; 10–20 ng/μl for cercariae and eggs), 2 μl DMSO (dimethyl sulfoxide) and 17 μl H_2_O. All PCRs were performed in an MJ PTC-100 thermal cycler with initiation at 94 °C for 5 min, followed by 35 cycles consisting of denaturation for 30 s at 94 °C, annealing at 56 °C for 30 s, extension at 72 °C for 3 min.

### Sequencing and sequence analyses

PCR products were obtained from at least two individual samples for each template (i.e. adults, metacercariae, cercariae and eggs) originating from different geographical localities. The PCR products (10 μl of each) were examined on a 1% agarose gel, stained with ethidium bromide, and visualized under UV light (Wealtec, Meadowvale Way Sparks, USA).

All the purified or gel-extracted amplicons were subjected to direct sequencing by automated sequencers using amplifying/flanking and internal primers (Table 2) by primer-walking in both directions (Macrogen Inc., Seoul, South Korea). Sequences (two from each sample) were aligned to obtain the final sequence for characterization. All sequences of *Opisthorchis* sp. BD2013 were identical, regardless of the life-cycle stage or locality.

The concatenated nucleotide and amino acid sequences of three protein-coding genes, i.e., *cob* + *nad*1 + *cox*1, were used to infer the pairwise genetic distances between 10 opisthorchiids (Table 3). These isolates included *Opisthorchis* sp. BD2013 and the reference sequences from Laos (JF739555), Vietnam (MF287777–MF287779) and Thailand (MF287780–MF287782). The genetic distances were inferred by pairwise analysis using the MEGA6.0 software, and the number of base substitutions per site was calculated by the most simplified method (uncorrected p-distance) [21].

**Table 3:** Summary data for complete mitochondrial genomes of species providing cytochrome *b* (*cob*), nicotinamide dehydrogenase subunit 1 (*nad*1) and cytochrome *c* oxidase subunit 1 (*cox*1) used in the phylogenetic analysis including *Opisthorchis* sp. BD2013 in ducks in Vietnam

Family/Species	Isolates/Strains	Country	GenBank ID	Reference
Opisthorchiidae				
*Opisthorchis* sp. BD2013	PC6aduBD	Vietnam	MF287762–MF287764	This study
*Opisthorchis* sp. BD2013	PM10aduBD	Vietnam^b^	MF287765–MF287767	This study
*Opisthorchis* sp. BD2013	PCmetaBD	Vietnam	MF287768–MF287770	This study
*Opisthorchis* sp. BD2013	PCcercaBD	Vietnam	MF287771–MF287773	This study
*Opisthorchis* sp. BD2013	PCeggBD	Vietnam	MF287774–MF287776	This study
*Opisthorchis viverrini*	na	Laos^b^	JF739555	[19]
	Binh Dinh 1	Vietnam^b^	MF287777–MF287779	This study
	Khon Kaen	Thailand^b^	MF287780–MF287782	This study
*Opisthorchis felineus*	Ust-Tula (Novosibirsk)	Russia^b^	EU921260	[16]
*Clonorchis sinensis*	Nam Dinh	Vietnam^c^	MF287783–MF287785	This study
	Guangdong	China^b^	JF729303	[19]
	na	South Korea^b^	JF729304	[19]
	Amur - Khabarovsk	Russia^b^	FJ381664	[16]
*Metorchis orientalis*	Heilongjiang	China^b^	KT239342	[22]
Heterophyidae				
*Haplorchis taichui*	na	Laos	KF214770	[24]
	Quang Tri 3	Vietnam	MF287786–MF287788	This study
*Metagonimus yokogawai*	na	South Korea	KC330755	
Fasciolidae				
*Fasciola hepatica*	Geelong	Australia	AF216697	[25]
*Fasciola gigantica*	Guangxi	China	KF543342	[26]
	Thua Thien-Hue	Vietnam	MF287789–MF287791	This study
*Fasciola* sp*.* (intermediate form)	GHL-Heilongjiang	China	KF543343	[26]
*Fasciolopsis buski*	Jiangxi	China	KX169163	[27]
	Ha Tay	Vietnam	MF287792–MF287794	This study
*Fascioloides magna*	Kokořínsko	Czech Republic	KU060148	[28]
Schistosomatidae				
*Schistosoma haematobium* ^a^	N10 Village	Mali	DQ157222	[29]

^a^Sequence used as the outgroup

^b^Sequences of the opisthorchiids used for pairwise genetic distance calculation (Tables 5 and 6)

### Phylogenetic analysis

#### Preparation of DNA sequences

Phylogenetic analysis using three mitochondrial protein-coding (*cob*, *nad*1, *cox*1) and two nuclear ribosomal (18S and 28S rDNA) genes was conducted to examine the taxonomic placement of *Opisthorchis* sp. BD2013 from ducks within the superfamily Opisthorchioidea. Sequences of trematode species/isolates of the Opisthorchiidae, Heterophyidae, Fasciolidae and Schistosomatidae (as the outgroup) were used. Summary data of species/isolates, mainly from the available complete mitochondrial genomes are presented in Table 3. Accession numbers for the target and reference 18S and 28S rDNA sequences are listed in Table 4. For *Opisthorchis* sp. BD2013, we decided to use only two sequences of adults, and one each from metacercariae, cercariae and eggs for phylogenetic analyses.

**Table 4:** Accession numbers of the reference 18S and 28S rDNA sequences and their species information used for phylogenetic analysis with those derived from *Opisthorchis* sp. BD2013 in ducks in the present study

Family/Species	18S rDNA GenBank ID (isolate)^b^	28S rDNA GenBank ID (isolate)^b^	Origin of sequences	Reference
Opisthorchiidae				
*Opisthorchis* sp*.*	MF077358 (PC6aduBD)^b^	MF110001 (PC6aduBD)	Vietnam	This study
	MF077359 (PCcercaBD)	MF110002 (PCcercaBD)	Vietnam	This study
	MF077360 (PCeggBD)	MF110003 (PCeggBD)	Vietnam	This study
	MF077361 (PCmetaBD)	MF110004 (PCmetaBD)	Vietnam	This study
	MF077362 (PM10aduBD)	MF110005 (PM10aduBD)	Vietnam	This study
*Opisthorchis viverrini*	HM004211 (SK)	HM004188 (SK);	Thailand	[30]
	JF823987 (THASK)	JF823990 (THASK)	Thailand	[17]
	MF077364 (PY2)	MF099792 (PY2)	Vietnam	GenBank
	MF077363 (BD1)	KY369165 (BD1)	Vietnam	GenBank
*Opisthorchis felineus*	MF077357 (Ust-Tula)	MF099790 (Ust-Tula)	Russia	GenBank
*Clonorchis sinensis*	JF823988 (VNM)	JF823989 (VNM)	Vietnam	[30]
	JF314770 (GD)	JF823989 (VNM)	China; Vietnam	GenBank; [30]
	MF077353 (NH)	MF099784 (NH)	Vietnam	GenBank
Heterophyidae				
*Haplorchis pumilio*	HM004194 (HpNP1)	HM004186 (HpNP1)	Thailand	[18]
	KX815125 (HPU8)	KX815125 (HPU8)	Vietnam	[12]
*Haplorchis taichui*	KX815126 (QT3)	KX815126 (QT3)	Vietnam	[12]
	HM004201 (NA3)	HM004187 (NA3)	Thailand	[30]
*Haplorchis yokogawai*	HM004207 (CP1)	HM004178 (CP1)	Thailand	[18]
	HM004208 (CP2)	KY369160 (An394)	Thailand; Vietnam	[12, 18]
*Procerovum varium*	HM004199 (PvNP1)	HM004182 (PvNP1)	Thailand	[30]
	MF077365 (HspND)	KY369161 (HspND)	Vietnam	GenBank; [12]
*Stellantchasmus falcatus*	HM004202 (VN1)	HM004174 (VN1)	Vietnam	[17]
	MF077366 (QN2)	KY369164 (QN2)	Vietnam	[12]
*Metagonimus takahashii*	HQ832629 (Mt3)	HQ832638 (Mt3)	Japan	[31]
*Metagonimus yokogawai*	HQ832630 (My1)	HQ832639 (My1)	Japan	[31]
*Metagonimus miyatai*	HQ832626 (Mm3)	HQ832635 (Mm3)	Japan	[31]
Fasciolidae				
*Fasciolopsis buski*	AY311386 (Vinh)	EU025870 (NA)	Vietnam	[32]
*Fasciola gigantica*	MF077354 (NB)	MF099787 (NB)	Vietnam	GenBank
*Fasciola hepatica*	MF077355 (Geelong)	MF099788 (Geelong)	Australia	GenBank
*Fascioloides magna*	EF051080	EU025872	United States	GenBank; [33]
Schistosomatidae				
*Schistosoma haematobium* ^a^	Z11976	AY157263	Mali	[34, 35]

^a^Sequence used as the outgroup

^b^Abbreviations for isolates are given in parentheses

Concatenated nucleotide sequences of mt protein-coding genes (*cob*, *nad*1, *cox*1) from adults, metacercariae, cercariae, and eggs of *Opisthorchis* sp. BD2013, and from additional taxa (available in GenBank; see Table 3) were imported into GENEDOC 2.7 (available at http://iubio.bio.indiana.edu/soft/molbio/ibmpc/genedoc-readme.html) and aligned for phylogenetic analysis. Additionally, the sequences of opisthorchiids were translated (using the echinoderm/flatworm mitochondrial genetic code: translation Table 9 in GenBank), and the deduced amino acid sequences were aligned for pairwise genetic distance analysis.

DNA sequences of 18S rRNA and 28S rRNA genes (listed in Table 4) were aligned separately using GENEDOC 2.7. The sequences were trimmed at both ends to the shortest length of the representative sequences. For 18S rDNA, in this study, the final alignment was 2005 nucleotides (nt) long of which 87 nt positions were trimmed at 5′ end and 114 nt at 3′ end, leaving 1804 characters for analyses. For 28S rDNA, the final alignment was1449 nt long of which 122 nt positions were trimmed at 5′ end and 123 nt at 3′ end, leaving 1202 characters for analyses. The two sequences were then concatenated as indicated in Table 4, preferably from the same strains/isolates. The concatenated 18S + 28S rDNA sequences representing species/isolates were imported into GENEDOC 2.7 and phylogenetic analysis and tree construction were done by MEGA6.0 [21].

#### Phylogenetic reconstruction

The alignments of the concatenated nucleotide (*cob*, *cox*1, *nad*1) and 18S +28S sequences, respectively, were trimmed to the length of the shortest sequence and imported into the MEGA 6.06 software [21]. Maximum likelihood (ML) analyses were performed in each case. For DNA sequences, we used the general time-reversible model of evolution with gamma distributed rate heterogeneity and a proportion of invariant sites (GTR + Γ + I). This model was given the best Bayesian information criterion score by MEGA. For amino acid sequences, the Jones-Taylor-Thornton (JTT) model with uniform rates and Nearest-Neighbor-Interchange (NNI) method was used. The confidence in each node was assessed using 1000 bootstrap resamplings [21].

## Results

### Mitochondrial *cob*, *nad*1, *cox*1 and genetic distances among opisthorchiid species/sequences

For *Opisthorchis* sp. BD2013, lengths of the complete *cob*, *nad*1 and *cox*1 genes were 1110, 903 and 1551 nucleotides, respectively. Among opisthorchiid species, *cob* genes ranged in length from 1110 to 1116 nt, and *cox*1 genes were 1551 to 1563 nt in length. The primer pairs U18SF/U18SR were used for obtaining major fragments of ribosomal 18S and U28SF/U28SR for 28S rDNA.

Nucleotide and amino acid pairwise comparisons of the concatenated mt genes among ten opisthorchiid isolates/species are presented in Tables 5 and 6. The concatenated *cob* + *nad*1 + *cox*1 nucleotide sequences of *Opisthorchis* sp. BD2013 differed at 14.4–14.5% of nucleotide sites and 10.3–10.6% of amino acid positions from the reference sequences of *O. viverrini* (Vietnam, Thailand and Laos isolates) [19]; 17.9–18.2% for nucleotides and 13.3–13.7% for amino acids from *C. sinensis* (Russia, China, South Korea and Vietnam isolates); 18.1% (nucleotides) and 13.7% (amino acids) from *O. felineus* (a Russian isolate) [16] and 15.4% (nucleotides) and 11.6% (amino acids) from *Metorchis orientalis* (China isolate) [23].

**Table 5:** Pairwise genetic distances (%) between *Opisthorchis* sp. BD2013 sample from ducks in Vietnam and the sequences for *O. viverrini*, *Clonorchis sinensis*, *O. felineus* and *Metorchis orientalis* of the concatenated mitochondrial genes *cob*, *nad*1 and *cox*1

	Species	GenBank ID	1	2	3	4	5	6	7	8	9	10
1	*Opisthorchis* sp. BD2013 (PM10aduBD/Vietnam)	MF287767	–									
2	*O. viverrini* (Binh Dinh 1/ Vietnam)	MF287779	14.4	–								
3	*O. viverrini* (Khon Kaen/ Thailand)	MF287782	14.5	0.4	–							
4	*O. viverrini* (Laos)	JF739555	14.4	0.5	0.7	–						
5	*C. sinensis* (Amur-Khabarovsk/Russia)	FJ381664	17.9	18.1	18.1	17.9	–					
6	*C. sinensis* (Guangdong/ China)	JF729303	18.0	18.1	18.1	17.9	0.4	–				
7	*C. sinensis* (South Korea)	JF729304	18.2	18.2	18.3	18.0	0.5	0.3	–			
8	*C. sinensis* (Nam Dinh/ Vietnam)	MF287784	18.0	18.1	18.2	18.0	0.5	0.5	0.6	–		
9	*O. felineus* (Ust-Tula/ Russia)	EU921260	18.1	18.8	18.9	18.7	15.4	15.6	15.8	15.5	–	
10	*Metorchis orientalis* (Heilongjiang/China)	KT239342	15.5	13.7	13.7	13.5	17.0	17.2	17.2	17.0	16.8	–

**Table 6:** Pairwise genetic distances (%) between *Opisthorchis* sp. BD2013 sample from ducks in Vietnam and *O. viverrini*, *Clonorchis sinensis*, *O. felineus* and *Metorchis orientalis* of the concatenated mitochondrial amino acid sequence of *cob*, *nad*1 and *cox*1

	Nucleotide sequences	Accession No.	1	2	3	4	5	6	7	8	9	10
1	*Opisthorchis* sp. BD2013 (PM10aduBD/Vietnam)	MF287767	–									
2	*O. viverrini* (Binh Dinh 1/ Vietnam)	MF287779	10.6	–								
3	*O. viverrini* (Khon Kaen/ Thailand)	MF287782	10.6	0.5	–							
4	*O. viverrini* (Laos)	JF739555	10.3	0.6	0.6	–						
5	*Clonorchis sinensis* (Amur-Khabarovsk/Russia)	FJ381664	13.3	12.4	12.4	12.4	–					
6	*C. sinensis* (Guangdong/ China)	JF729303	13.5	12.8	12.8	12.8	0.3	–				
7	*C. sinensis* (South Korea)	JF729304	13.7	12.7	12.7	12.7	0.3	0.2	–			
8	*C. sinensis* (Nam Dinh/ Vietnam)	MF287784	13.6	12.6	12.6	12.6	0.4	0.8	0.8	–		
9	*O. felineus* (Ust-Tula/ Russia)	EU921260	13.7	13.8	13.9	13.9	9.3	9.7	9.7	9.5	–	
10	*Metorchis orientalis* (Heilongjiang/China)	KT239342	11.6	8.8	8.8	8.7	9.8	10.2	10.2	10.1	11.0	–

Within each opisthorchiid taxon, pairwise genetic distances were small, only 0.4–0.7% for nucleotides and 0.5–0.6% for amino acids within *O. viverrini*; 0.3–0.6% (nucleotides) and 0.2–0.8% (amino acids) within *C. sinensis*. *Opisthorchis* sp. BD2013 in ducks differs from *O. viverrini* by more than 10%, a figure comparable to those separating species within the genus *Opisthorchis* and the family Opisthorchiidae (Tables 5 and 6).

### Phylogenetic analysis

#### Phylogenetic reconstruction based on the complete *cob* + *nad*1 + *cox*1 amino acid sequences

**Fig. 1:**
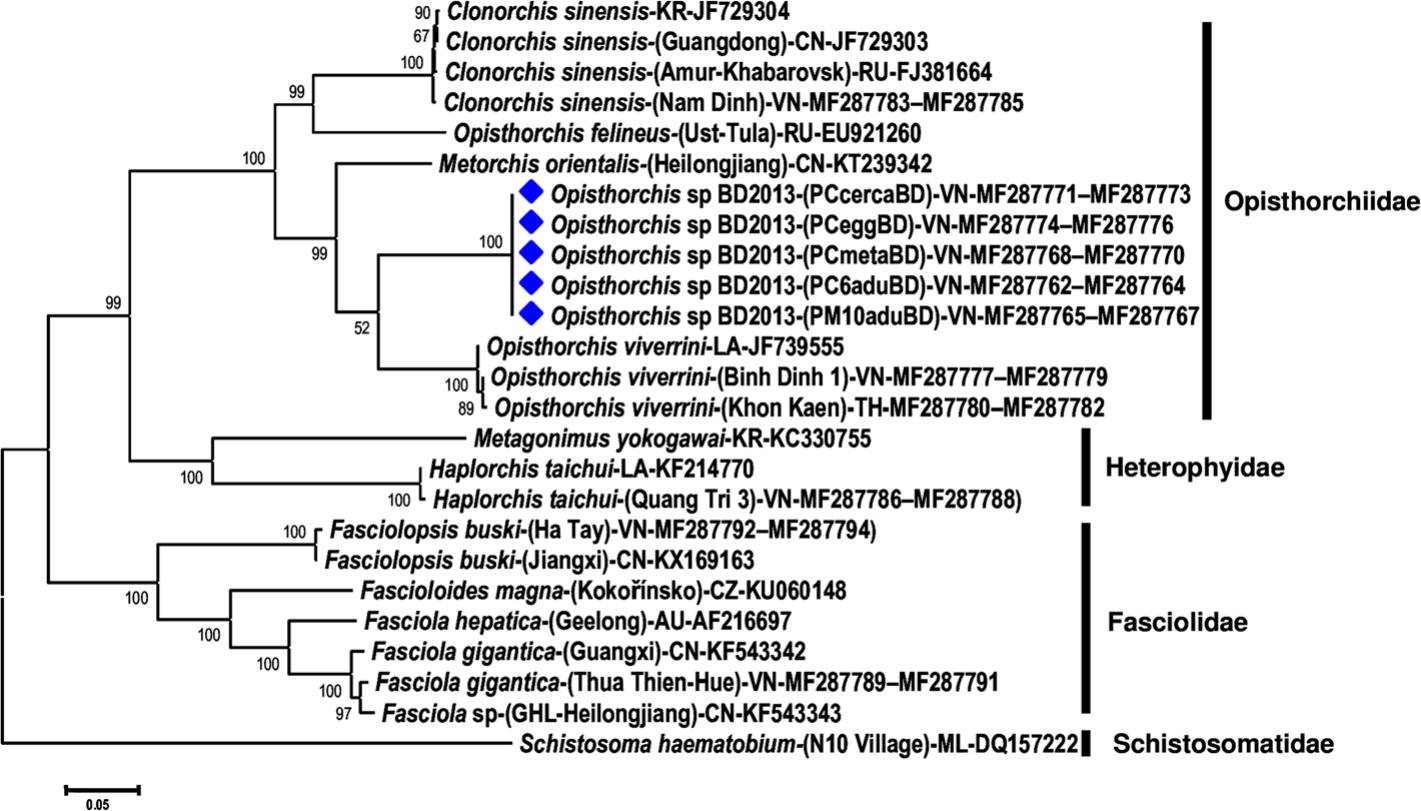
Phylogenetic tree for *Opisthorchis* sp. BD2013 (indicated by diamond symbol) and other opisthorchiids and representative trematodes from 4 families, the Opisthorchiidae, Heterophyidae, Fasciolidae and Schistosomatidae (the latter used as an outgroup), based on concatenated nucleotide sequences of complete cytochrome b (*cob*), nicotinamide dehydrogenase subunit 1 (*nad*1) and cytochrome *c* oxidase subunit 1 (*cox*1) genes. Phylogenetic reconstruction was performed using maximum likelihood analysis with the general time-reversible model with a gamma distributed rate heterogeneity and a proportion of invariant sites (GTR + Γ+ I) in the MEGA6.06 software package. Support for each node was evaluated using 1000 bootstrap resamplings [21]. The scale-bar indicates the number of substitutions per site. Accession numbers (where available) are given at the end of each sequence name. Isolates/geographical localities are given in parentheses (if available). Country abbreviation codes (2-letter) given prior to the accession numbers: AU, Australia; CN, China; CZ, Czech Republic; KR, Korea; LA, Lao PDR; RU, Russia; TH, Thailand; VN, Vietnam

A phylogenetic tree was constructed from 25 nucleotide sequences inferred from complete *cob* + *nad*1 + *cox*1 of 13 trematode species belonging to 4 families with *Schistosoma haematobium* of the Schistosomatidae as the outgroup (Table 3, Fig. 1). The superfamily Opisthorchioidea in this study comprises the Heterophyidae and Opisthorchiidae (no appropriate sequences from the third family, Cryptogonimidae, were available), with the strong nodal support of 99%, clearly separate from the family Fasciolidae. The *Opisthorchis* sp. BD2013 clade was placed as a sister of *O. viverrini* from Thailand, Vietnam and Laos. The genus *Opisthorchis* appeared as paraphyletic with respect to *C. sinensis*, *O. felineus* and *M. orientalis* (Fig. 1).

#### Phylogenetic reconstruction based on partial 18S + 28S sequences

**Fig. 2:**
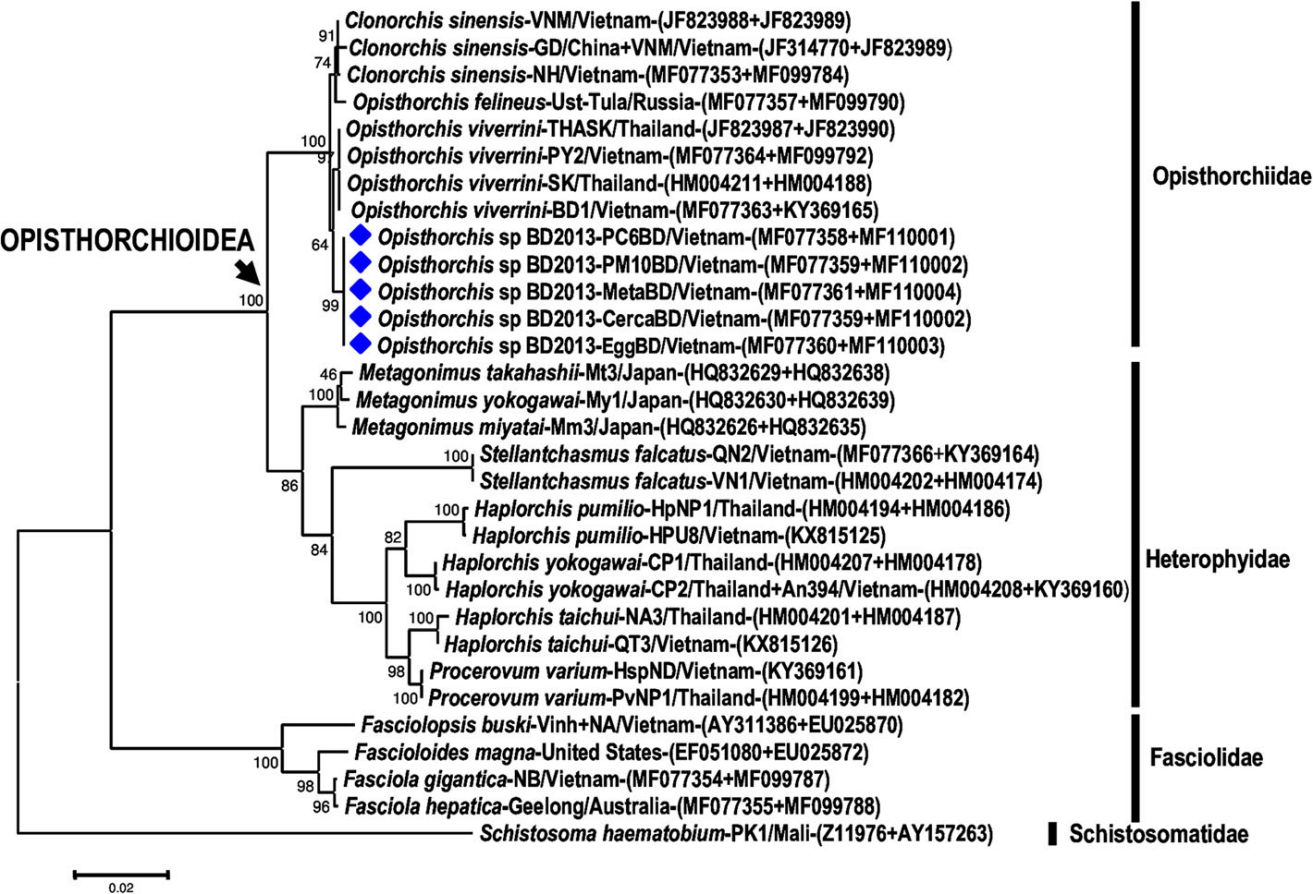
Phylogenetic tree for *Opisthorchis* sp. BD2013 (indicated by diamond symbol) and other opisthorchiids and representative trematodes from 4 families, the Opisthorchiidae, Heterophyidae, Fasciolidae and Schistosomatidae (the latter used as the outgroup), based on combined nucleotide sequences of the nuclear small ribosomal subunit (18S rDNA) and large ribosomal subunit (28S rDNA). Phylogenetic reconstruction was performed using maximum likelihood analysis with the general time-reversible model and a gamma distributed rate heterogeneity and proportion of invariant sites (GTR + Γ+ I) in the MEGA6.06 software package. Support for each node was evaluated using 1000 bootstrap resamplings [21]. The node for the superfamily (infraorder) Opisthorchioidea is indicated by an arrow. The scale-bar indicates the number of substitutions per site. Accession numbers are given at the end of each sequence name. Isolates or geographical localities and country isolated are given in the between (if available)

Five concatenated 18S + 28S sequences of Vietnamese *Opisthorchis* sp. BD2013 (from eggs, cercariae, metacercariae and adults) were aligned with 26 available sequences representing 17 trematode species of the Opisthorchiidae, Heterophyidae, Fasciolidae and Schistosomatidae (outgroup) (Table 4). The nuclear ribosomal dataset from the Opisthorchioidea included available sequences of the 18S + 28S of 12 taxa only from the Opisthorchiidae and Heterophyidae (data from the Cryptogonimidae were not available). The combined length of alignment in use was between 2940 and 2960 nt. The inferred phylogenetic tree (Fig. 2) again placed *Opisthorchis* sp. BD2013 in a sister position with *O. viverrini* from Thailand and Vietnam. Again, the genus *Opisthorchis* appeared as paraphyletic. Monophyly of the superfamily Opisthorchioidea was strongly supported (Fig. 2).

## Discussion

In this study, we used two concatenated datasets to infer the molecular phylogenetic position of *Opisthorchis* sp. BD2013 (formerly named “*Opisthorchis viverrini*-like” or as *O. parageminus* by several authors). We did not have samples of *O. lobatus* [17] and the so-called *O. parageminus* [8, 9] for analysis in the present study, therefore, we were not able to establish the relationship between *Opisthorchis* sp. BD2013 and these species.

The genus *Opisthorchis* is very large [7], but relevant sequence data are limited to only a few species. It was necessary to determine whether *Opisthorchis* sp. BD2013 from ducks is distinct from *O. viverrini*, a zoonotic liver fluke known to infect and to cause cholangiocarcinoma in humans [23]. The data presented in this study strongly imply that the two are distinct species. The sister-species relationship demonstrated between *Opisthorchis* sp. BD2013, and *O. viverrini* might simply be because *O. felineus* is the only other member of the genus for which data are available. *Opisthorchis felineus* renders *Opisthorchis* paraphyletic in our trees, indicating that much systematic work remains to be done in the Opisthorchiidae. A further unresolved question is the relationship between *Opisthorchis* sp. BD2013 and *O. parageminus*. Both were found in ducks in Vietnam, but some morphological differences seem to exist [11]. At this stage, we prefer to leave the question open, pending future morphological and molecular work.

Our previous phylogenetic analysis using short sequences of ITS2 and *cox*1 revealed close affinities between *O. viverrini*, *O. lobatus* and *Opisthorchis* sp. BD2013 [5]. In the current study, we are unable to resolve the status of *O. lobatus* compared to *Opisthorchis* sp. BD2013 and other opisthorchiids*.*

## Conclusions

Based on mitochondrial *cob* + *nad*1 + *cox*1 and ribosomal 18S + 28S rRNA sequence analyses, *Opisthorchis* sp. BD2013 was distinct from *O. viverrini*, although the two species are closely related. The genus *Opisthorchis* itself appears as paraphyletic. Data from additional *Opisthorchis* species are vital to create a phylogeny with higher resolution within *Opisthorchis* and the Opisthorchiidae*.*

## Abbreviations

*cob*: cytochrome *b*
*cox*1: cytochrome *c* oxidase subunit 1
MEGA: Molecular Evolutionary Genetics Analysis
ML: maximum likelihood
mt: mitochondrial
*nad*1: nicotinamide dehydrogenase subunit 1
rTU: ribosomal transcription unit
